# Access and benefits sharing of genetic resources and associated traditional knowledge in northern Canada: understanding the legal environment and creating effective research agreements

**DOI:** 10.3402/ijch.v72i0.21351

**Published:** 2013-08-05

**Authors:** Janis Geary, Cynthia G. Jardine, Jenilee Guebert, Tania Bubela

**Affiliations:** School of Public Health, University of Alberta, Edmonton, Canada

**Keywords:** northern Canada, access and benefits sharing, genetic resources, research agreements

## Abstract

**Background:**

Research in northern Canada focused on Aboriginal peoples has historically benefited academia with little consideration for the people being researched or their traditional knowledge (TK). Although this attitude is changing, the complexity of TK makes it difficult to develop mechanisms to preserve and protect it. Protecting TK becomes even more important when outside groups become interested in using TK or materials with associated TK. In the latter category are genetic resources, which may have commercial value and are the focus of this article.

**Objective:**

This article addresses access to and use of genetic resources and associated TK in the context of the historical power-imbalances in research relationships in Canadian north.

**Design:**

Review.

**Results:**

Research involving genetic resources and TK is becoming increasingly relevant in northern Canada. The legal framework related to genetic resources and the cultural shift of universities towards commercial goals in research influence the environment for negotiating research agreements. Current guidelines for research agreements do not offer appropriate guidelines to achieve mutual benefit, reflect unequal bargaining power or take the relationship between parties into account.

**Conclusions:**

Relational contract theory may be a useful framework to address the social, cultural and legal hurdles inherent in creating research agreements.

Research in northern Canada focused on Aboriginal peoples has historically benefited academia with little consideration for the people being researched or the integration of Indigenous or traditional knowledge (TK). In the past, it was not uncommon for researchers to take knowledge or data and misrepresent Aboriginal peoples, collect human tissue samples for one purpose and use them for another, or collect other biological samples to conduct research on the samples without the consent of the community. In other words, as acknowledged in a 2012 report by the Canadian National Collaborating Centre for Aboriginal Health (NCCAH), research has historically failed to benefit Aboriginal peoples. The conclusions of this damning report were alarming, highlighting that research has been harmful, often counterproductive to improving health, and insensitive, intrusive and exploitive to Aboriginal peoples (1).

The imbalance of power in research has been described as colonising (2) because Aboriginal peoples are systematically excluded from a process of creating knowledge that does not recognise Aboriginal TK or world views because it is defined predominantly by Western thought (3). However, incorporating TK into a Western scientific research paradigm is a complex undertaking. Adding to the complexity is that the term TK has a multiplicity of meanings, including knowledge systems, innovations and customary laws or practices (4). Furthermore, TK is not static. It is considered traditional not because it is old, but because of “the way it is acquired and used” (5) (p. 73). It encompasses not only factual knowledge about the environment, land and species that live in it, but also values about using the environment (6) and the framework of observations and experiences which form the body of knowledge (7). Unlike Western forms of knowledge, TK is often specific to a place, held collectively by members of a community, and transmitted orally (8). It can be understood as the body of knowledge that has allowed the cultural group(s) holding it to adapt to changes in their environment (9). The complexity of TK makes it difficult to develop mechanisms to preserve and protect it from misuse or misappropriation. However, as outside groups become interested in using TK or materials with associated TK, it becomes increasingly important to develop these mechanisms.

The focus of this article is on genetic resources,^1^ which fall into the category of materials with associated TK. Genetic resources may have commercial value and therefore outside groups are becoming increasingly interested in gaining access to them. If a knowledge holder grants outsiders access to genetic resources, any benefits that arise should be fairly shared with the knowledge holder. While monetary benefits are appropriate in some circumstances, our understanding of benefits must be broader, incorporating knowledge exchange with communities; implementing mechanisms to protect cultural heritage and artefacts; developing infrastructure; and/or providing training opportunities.

This article addresses access to and use of genetic resources and associated TK in the context of the historical power imbalances that persisted in research relationships in the Canadian North. First, we describe why research on genetic resources is of growing relevance. Next, we describe the legal framework for research using genetic resources. This includes the international legal framework that gives rise to national obligations, Aboriginal rights protected in Canadian law, as well as the policies and guidelines of Canadian granting agencies and institutions. This legal backdrop influences the environment for negotiating research agreements between northern communities and Canadian institutions. However, other conflicting pressures also impact these negotiations, including the cultural shift of universities since the 1980s towards research with commercial value. As universities increasingly seek to commercialise the products of research, mechanisms that ensure there is fair and equitable access and benefits sharing (ABS) of genetic resources become increasingly critical. This article therefore culminates in a theoretical and practical discussion of research agreements that respect and meet the needs of research partners, while acknowledging the power-imbalance inherent in their negotiation. This article concludes with recommendations for developing research agreements in Canada's north.

## The growing importance of genetic resources in the North

The narrative of a powerful corporation exploiting tropical biodiversity and local knowledge to produce new cosmetic or medicinal products, depicted in popular fiction such as the 1992 film *Medicine Man*, is familiar to many. While a simplified representation of the issues, movies like *Medicine Man* stimulate international debates about the commercial exploitation of genetic resources and associated TK. This activity often referred to as “bioprospecting” by proponents, and “biopiracy” by detractors (9).

While images of expansive Arctic tundra do not, at first glance, fit the familiar description of lush biodiversity overflowing with potential scientific discoveries, there is a growing interest in the genetic resources of the North (11). Given its harsh and often extreme environment, the Arctic is home to potentially valuable genetic resources that could be developed into various biotechnologies. A report by the United Nations University – Institute of Advanced Studies outlined the main areas with potential for biotechnology research in the Arctic (12). The interest in the Arctic is broad and includes enzymes for industrial processes, bioremediation, anti-freeze proteins, dietary supplements and pharmaceuticals. Microbes are of particular interest, as microbial diversity in the Arctic is greater than previously assumed (13). As a result, the Marine Natural Products Lab at the University of Prince Edward Island and Nunavut Tunnagavik have negotiated a potentially precedent setting ABS agreement for microbial bioprospecting in Nunavut. Researchers started expeditions in 2011 to collect marine mud and specimen samples in the search for new natural products such as beauty/health products, nutraceuticals and pharmaceuticals (14). Canadian and international researchers are also bioprospecting cold-adapted enzymes from microbes and mosses in Canada's Arctic (15,16).

In addition to research that is oriented towards developing commercialisable products, there are many projects in the Canadian Arctic that focus on TK associated with genetic resources. Communities are especially interested in documenting and preserving TK in relation to traditional medicines because of concerns about the impacts of climate change on their accessibility (17). For instance, the Gwich'in Ethno-Botany Study being undertaken by the Gwich'in Renewable Resources Board aims to systematically collect and document information about plant use, mainly those used for traditional medicines (18). A Health Canada program funded several traditional medicine projects that resulted in workshops, books, maps and a herbarium (17). The Acho Dene Koe First Nation in the Northwest Territories (NWT) mapped medicinal territory and incorporated the locations of plants into their existing mapping system (19). The Ross River First Nation in Yukon created a database of important plants, including maps of where they grow, as well as a book: *Gu None’: Ethnobotany of the Ross River Dena* (20).

Given the increasing interest in searching for commercialisable products in Canada's Arctic, and the importance of TK associated with genetic resources such as medicinal plants, it is important for researchers and communities to understand the relevant laws and guidelines. This legal framework informs how genetic resources and associated TK may be accessed, and how benefits resulting from research should be shared and managed.

## Legal framework for research using genetic resources

### The international legal framework

Much of the world's genetic resources are subject to the Convention on Biological Diversity (CBD). Canada was one of the first of 168 countries to ratify this international treaty in 1992, while the influential United States has still yet to do so (21). The objectives of the CBD are “the conservation of biological diversity, the sustainable use of its components and the fair and equitable sharing of the benefits arising out of the utilization of genetic resources” (10). Article 15 of the CBD grants the rights to genetic resources to the sovereign state, not to Indigenous communities, and presumes those states will make genetic resources accessible for research (10). The interests of states and their Indigenous peoples are not necessarily aligned. In Article 15(7) of the CBD, countries are asked to take appropriate measures that aim at “sharing in a fair and equitable way the results of research and development and the benefits arising from the commercial and other utilisation of genetic resources” with the Indigenous communities they have entered into an agreement with (10). Canada has also endorsed the non-binding *United Nations Declaration on the Rights of Indigenous Peoples* (22). However, Canada has yet to implement national provisions related to ABS of genetic resources. In 2009, Environment Canada sought feedback on ABS policy in Canada, based on a discussion paper which presented 3 options for addressing TK in ABS policies in Canada ranging from voluntary mechanisms to specific legislation (23), but points out that under the CBD Canada is not obliged to do so and questions whether or not it should (23). There has been no evident progress since then at the national level.

As a supplement to the ABS provisions in the CBD, the Nagoya Protocol on Access to Genetic Resources and the Fair and Equitable Sharing of Benefits Arising from their Utilization to the Convention on Biological Diversity was adopted on October 29, 2010 (24). The protocol clarifies and emphasises the importance of ensuring that genetic resources and associated TK are accessed and utilized in a fair and equitable way. The basic principles for gaining access require obtaining prior informed consent of TK holders as well as negotiating agreements with mutually agreed terms (25). Article 8(1) of the protocol suggests simplified procedures for those who wish to access genetic resources for non-commercial use (24). International discussions on these issues continue in an attempt to clarify the language and implications of the protocol. It has not yet entered into force, and will not do so until 90 days after the 50th country ratifies. As of March 2013, while 92 countries have signed on to the protocol, only 15 have ratified (21). Thus, the protocol remains an aspirational document that represents an internationally negotiated compromise on ABS and genetic resources between the interests of groups such as Like Minded Megadiverse Countries (LMMC) and the industrialised world.

### Agreements negotiated between the Canadian government and Aboriginal peoples

At the international level there is a lack of clarity on appropriate provisions for ABS and TK. Domestically, Canada has been slow to implement ABS provisions in the CBD, with the last concrete action being a consultation report released in 2005 (26). There are several reasons why Canada has not advanced ABS implementation. Stakeholders have yet to reach consensus on how to implement ABS policies, Indigenous peoples in Canada have voiced concerns over ABS policies and the Nagoya Protocol (27), and the government has faced little pressure from stakeholders for implementation of an ABS framework. Despite these factors, the interests of Aboriginal peoples and their TK are protected through the Canadian Constitution and other domestic policies. The Constitution Act (28) recognises and affirms Aboriginal and treaty rights of Indian (sic), Inuit and Métis peoples, which has been interpreted to include Aboriginal rights, Aboriginal land title and treaty rights. The Supreme Court of Canada (SCC) addressed and elaborated what Aboriginal rights encompass in several cases (29–32). They include rights to traditional activities, such as hunting and fishing, and are a key factor in ensuring the continued protection and transmission of TK (26).

The SCC has ruled that Aboriginal rights can be justifiably infringed in certain circumstances that meet 2 conditions: the government must have a valid legislative objective, supported by evidence; and the infringement must be in keeping with the government's fiduciary obligations (29). This latter obligation is part of a duty of the Crown through the Government of Canada to consult and accommodate Aboriginal peoples. Such consultation ensures Aboriginal peoples are represented when their rights are affected, even in the absence of self-government and land claim agreements. The SCC has also ruled that the government must consider additional elements when infringing an Aboriginal right, including: giving priority to accommodating the Aboriginal right; ensuring that the right is minimally infringed; that it adequately consults with any affected communities; and that compensation has been provided where required.

While the duty to consult applies broadly to situations when Aboriginal rights may be impacted, governments have also used legislation to impose a duty to consult. Examples of legislated mandates to consult Aboriginal peoples are found throughout federal, provincial and territorial laws in areas as diverse as the environment, the management of protected areas, conservation, development, food and drugs, health and social services, employment and corrections (26). The implication for researchers wishing to work with Aboriginal groups or within their territories is that there are legal requirements that must be met before they can do so. In most circumstances, researchers must receive permits from their Aboriginal partners.

### Canadian policies and guidelines for the ethical conduct of research with Aboriginal peoples

Aboriginal peoples in Canada have successfully advocated for greater control over what research is allowed in their communities, what happens with the data that are collected, and for equal respect of traditional/local knowledge systems alongside Western science (33). Formal structures for overseeing research with Aboriginal communities exist at the community level, in Territorial legislation, and through Federal granting agencies’ rules.

Some self-governing Aboriginal communities have established local review procedures for research. For example, the Vuntut Gwitchin First Nation has an extensive history of coordinating research in their territory (34). All researchers who wish to conduct work in their territory must partner with the community, be subject to a local ethics review, and agree to deposit all documentation and reports resulting from the research with the Vuntut Gwitchin First Nation's Heritage department.

Territorial governments have passed legislation to govern research (35–37) through licensing and the establishment of institutes such as the Aurora Research Institute in the NWT and the Nunavut Research Institute in Nunavut (33). Research in the Yukon is reviewed under the Association of Canadian Universities for Northern Studies (ACUNS) (38).

The Canadian Institutes of Health Research (CIHR), Canada's major national funding agency for health research, recommends that academic researchers who are applying for funds to work with Aboriginal peoples submit research agreements with their funding applications (8). CIHR, along with Canada's 2 other major national funding agencies (collectively known as the Tri-Council), have drafted specific ethical guidelines that researchers working with Aboriginal peoples need to follow. The guidelines include, among other things, requiring community engagement, advising research partners to set out terms of the relationship in a research agreement and including of intellectual property rights^2^ (IPRs) provisions where applicable, as well as licensing and sharing of royalties.

In addition to respecting the legally mandated rights of Aboriginal peoples, publicly funded institutions like universities have an implied obligation to pursue research that is only in the best interests of Aboriginal peoples. More broadly, universities have an obligation to fulfil their social mission to pursue knowledge and research that benefits all of society. Unfortunately, this mission is often at odds with increasing pressures to commercialise research (39).

## Shift of publicly funded research institutions towards research with commercial value

Universities have traditionally existed to benefit society through the provision of public goods and services. However, since the 1980s there has been a notable shift in universities towards the commercialisation or the rapid translation of research outputs. Researchers are actively encouraged to pursue patents, license technologies and create spin-off companies (40). The pressure to commercialise comes from governments and funding agencies eager to see a return on investment in research. Universities also perceive that commercialisation will result in direct financial benefits in an era of declining financial support for tertiary education (41). Most research-intensive universities now house a technology transfer office (TTO) tasked with managing the intellectual property (IP) generated within the institution (42). TTOs protect the interests of the university and/or its researchers, depending on whether IP is owned by the university or the researcher(s). This focus on commercialisation and IPRs has been criticised for narrowing the social mission of universities (43) and damaging public trust in these institutions as they pursue commercial goals (44).

In the context of Aboriginal research partnerships, the institutional focus on IPRs is even more problematic. The nature of TK and the systems that preserve and transmit it make applying IPR to TK difficult. IPR protection is granted for a limited time period on the condition that knowledge will be deposited into the public domain. Once the IPR expires, the knowledge holder no longer has control over it (9). For example, patent protection lasts for 20 years after which the invention enters the public domain. However, such limited protection is inconsistent with TK systems that customarily pass knowledge from one generation to the next, requiring perpetual protections, and does not equate to the public domain. This is well-captured in the following statement by the Saami Council: “Indigenous peoples have rarely placed anything in the so called “public domain”, a term without meaning to us … the public domain is a construct of the IP system and does not take into account domains established by customary indigenous laws” (45).

However, in some circumstances, it may be appropriate to form partnerships between researchers, their institutions and TK holders for the potential commercial exploitation of genetic resources. In such cases, the focus on IPRs and commercialisation in research institutions should not interfere with the building of strong partnerships based on mutual benefit. Therefore, it is essential to develop mechanisms to ensure that access to genetic resources with or without associated TK is accompanied by the fair and equitable sharing of benefits. This can only be accomplished through transparent negotiation processes that result in non-exploitative and mutually satisfactory research agreements, which reflect the spirit of the international treaties and the Canadian legal framework that protects the interests of Aboriginal peoples.

## Research agreements

### Challenges in developing research agreements

Despite the requirement to create research agreements, several issues make forming these agreements difficult and complicated. Research partners may have a different understanding of agreements, especially legal contracts, and their negotiation capacity may be uneven. Agreements may be bound by not only explicit terms in the document, but also implied terms such as good faith. Good faith means that parties must regard each other's interests and not act in a manner “that is contrary to community standards of honesty, reasonableness or fairness” (46). The relationships may even be fiduciary, where one partner is bound to act in the interest of the other, possibly even at the cost of their own self-interest (47). Partnerships may bring together different legal systems, which may not be formally recognised in each jurisdiction. Canadian researchers, who operate under the Canadian common law system which values individual rights, may find themselves partnering with indigenous groups who have customary legal systems that tend to emphasise communal rights (48). For example, the notion of land ownership to Inuit people, who view themselves as not simply owners but inhabitants who must share the land and respect the spirit of the land, is different from the Western perspective (49).

The social, cultural and legal hurdles to creating research agreements are exacerbated by a lack of clear guidance on what should be included in agreements and how these should be negotiated. In addressing how to create and use agreements to formalise these relationships, which often evolve over time, there is value in seeking guidance from contract theory. While not all research agreements would be considered legally enforceable, contract theory offers a body of work that can be used to help understand the development of research agreements that reflect the concepts of reciprocity and mutual benefit.

### Rationale for using relational contract theory as a framework for research agreements

Documents and guidelines exist on how to draft research agreements (50,51). Guidelines suggest that simply using template agreements is of limited value because the agreement “must embody, specifically, the deal that has been struck between the parties, and a good deal for all parties will depend on the purpose of the deal and the context in which the deal takes place” (51). However, there is limited theoretical and empirical guidance on how to create written agreements that account for the special considerations needed in Aboriginal research partnerships. In each partnership, partners need to determine for themselves what provisions might help increase the long-term stability of the partnership or help ensure mutual benefit, while recognising historical imbalances of power. Therefore, proposing a framework to guide the development of research agreements with Aboriginal partners with the goal of seeking mutual benefits is useful. This article proposes a framework that builds on relational contract theory, which enables successful long-term partnerships and explicitly addresses context and power structure.

Contracts, simply put, are promises that the law will enforce about the exchange of goods and services of value. In the early study of contracts, exchanges were assumed to be one-time events where each party attempted to maximise their own benefits; contracts were developed as tools to limit each party's ability to create benefit for themselves at the expense of the other (52). More recently, contract scholars have criticised the overly simplistic account of contracts as tools to limit self-maximisation of benefits because this view fails to account for the context of consensual exchanges (53). The realisation of the importance of contractual context gave rise to neoclassical contract law (54). Neoclassical contract law, while also assuming that parties will act out of self-interest to maximise benefits, integrates the context of the exchange into a deeper understanding of the contract (53). In neoclassical contract theory, context is largely viewed in terms of the relationship between the specific parties entering into a contract.

Relational contract theory is a further evolution of thought to recognise the importance of *societal* context and that all exchanges occur within relationships, many of which extend over long-periods of time (55). Recognising the societal context of research relationships between Aboriginal peoples and academics is an important step in developing effective research agreements. The classical view of contracts is not appropriate for these research agreements because it ignores the long-term nature of partnerships, and, by not accounting for societal context, it assumes that the cultural background of all parties is uniform. Relational contract theory, on the other hand, specifically applies to long-term partnerships, and can account for the societal context of research collaborations with Aboriginal peoples which have largely been shaped by the negative history of such collaborations.

The term “relational contract theory” was first used by Ian MacNeil in 1974 (56). The core of MacNeil's theory is a set of standard behaviors for contractual relationships, which he terms common norms. Common norms are those behaviors included in all contractual relationships, without which the relationship would fail (57). He then articulates an additional set of norms essential for highly relational and often long-term contracts, called the relational norms (58). Finally, he distinguishes between the “living contract”, which is the actual relationship between the parties, and the “contracts at law” which are the legal tools or provisions that govern the relationship (59). This latter distinction is important because it is impossible to describe the complete relationship within the legal document – the contract – especially for long-term relationships. Thus in summary, MacNeil distinguishes between the formal provisions of the contract and the essence of the long-term relationship, which the contract incompletely reflects.

## Applying the norms of relational contract theory to research agreements

The norms of relational contracts can be usefully applied to Aboriginal research collaborations and research agreements. The descriptions of these norms are based on several of MacNeil's foundational papers (56–58) as well as more recent works by other scholars (53,60).

*Norm 1*: Expectations of behavior are determined by a person's societal role, and each party's role in society dictates how they are expected to behave within the contractual relationship.

Parties planning to develop a research relationship need to address the expectations that they have of the societal role that the other represents. For example, research partners may have role-based expectations about who makes decisions about research design, what benefits are appropriate for each partner, where data are stored, or how they expect the other to behave. These expectations will inform how each group interprets the behavior of the other. A powerful example of the negative impact created by expected societal roles occurred in the high-profile battle between the Havasupai and Arizonan researchers. The researchers approached the community to take blood samples for what was explained to be diabetes research, a disease of particular concern to the community. However, the blood samples were also used for research projects which the Havasupai found offensive to their culture or stigmatising, such as studying the origin of their ancestors or mental illness (61,62). The resulting high-profile legal battle between the Havasupai and Arizonan researchers created a nation-wide mistrust of genetic researchers, despite the fact that a single research group in Arizona had misused blood samples (61,62). As a result, researchers who wish to collect blood samples from Indigenous groups in the United States will likely have to address the expectation that researchers will misuse such samples. To avoid such problems, discussions about the expectations of each party's roles should be integrated into the pre-ambles of agreements which should explain what the parties hope to achieve and why (63).

*Norm 2*: Each party expects that they will receive something in return for entering into the partnership.

It is important that both partnerships are mutually beneficial, and parties should receive benefits that they would not have had without the partnership. The benefits do not need to be the same, but they should be equitable. This ideal fits well with the spirit of the Nagoya Protocol, which emphasises not simply sharing, but fair and equitable sharing (24). Parties entering into a research partnership should outline what benefits they hope to receive from entering into the partnership. Discussing benefits also helps manage expectations of each party, which is important in building and maintaining good research relationships (63). Sometimes benefits may seem to be harmless to one party (e.g. academic researchers publishing in standard journals), but can actually be viewed as detrimental by the other party (e.g. colonial power structures that are reinforced through Western ideals about knowledge creation and dissemination) (2). If one party feels that the other's benefits might detract from their own, the parties can jointly determine a mechanism to achieve a more equitable distribution of benefits. To follow the abovementioned example, scientific publications could be written to acknowledge different knowledge systems or types of contributions to the research.

*Norm 3*: Parties will plan how to structure operations rather than define all potential exchanges.

When parties are entering into a research partnership, it is not possible to set the terms for every possible exchange that might happen over the course of the partnership. For that reason, the terms of a research agreement can set out how decisions will be made when they come up, rather than predict what decisions might need to be made in the future. Parties should articulate a representational management structure for the project and how operational decisions will be made. For example, parties may not know at the onset how many meetings they will need, but can plan for how they will decide when meetings will occur.

*Norm 4*: Parties consent to have their freedom limited by the other party.

Exercising choice in mutually beneficial research collaborations involves the sacrifice of certain opportunities by each party in exchange for opportunities that benefit the other party or are the least harmful to the other party. While this is difficult to work into research agreements, it is important for each party to recognise and understand that being involved in a research collaboration means consenting to limits on freedom of choice or action by the other party. For example, an academic researcher working alone would normally have freedom to give presentations at scientific meetings at his/her discretion. However, in collaborations other considerations arise which limit freedom of action, such as presenting to communities first or providing enough time for collaborators to approve or contribute content.

*Norm 5*: Without the capacity to change, contracts will break apart under pressures of change.

At the beginning of a research collaboration, there is limited information available to draft a research agreement. Research partners should assume that unanticipated events could impact the partnership over the course of their collaboration. Terms of research agreements should include mechanisms for flexibly adapting the research collaboration in response to changes to the research environment. For example, funding cuts in the government may impact research budgets, restricting planned activities, such as in-person meetings.

*Norm 6*: Parties will select behaviors that maintain the relationship.

Within the research collaboration, parties should select behaviors that help to maintain and preserve the relationship. Individual conflicts should be resolved for the sake of the overall relationship. Research agreements should always include terms for resolving conflicts, such as external mediation or even arbitration. Conflicts may be avoided if the contract clearly articulates expectations around co-management structures and communications (including the subject matter, means and frequency).

*Norm 7*: Expectations of the parties should be protected.

Each party will have expectations about what they will get from the partnership, based on what the other party has promised. Expectations should be the basis for assessing how parties receive restitution, and this is necessary for parties to be able to rely on each other, especially when there is no intention to create legal relationships. For example, the Havasupai expected that Arizonan researchers would only investigate diabetes. Despite vague consent forms, which allowed researchers to use the blood samples for other research, additional research was not consistent with Havasupai expectations, and a lengthy legal battle ensued to return the blood samples to the community (61). Assumptions should not be made about the other party's expectations. Instead, expectations should be discussed at the outset and throughout the relationship as it evolves.

*Norm 8*: The social relationship between parties creates and restrains power.

Power is the ability to make another do something that benefits the demanding party more than it benefits the other party. All contracts involve the creation and restraint of power. In research agreements, parties should discuss where power is inherent, such as funding structures which put all funding resources in the control of an academic researcher, and where the research agreement can be used to adjust and balance power in the research collaboration. For example, research directions and activities may be set through a joint management structure, while finances are held by one party.

*Norm 9*: Parties can pursue individual goals, but only by appropriate means within the context of the relationship.

In research collaborations, parties should define the means by which the ends may be achieved, and not only the ends themselves. For example, it would not be sufficient to understand that both parties intend to commercialise genetic resources resulting from collaborative research. Research agreements should include provisions that outline how the collaborators would make decisions about joint commercialisation, including ownership of IP and the distribution and form of benefits.

## Conclusions

Increasingly, international interest in genetic resources and associated TK in northern Canada raises the challenge of protecting and respecting Aboriginal interests (in genetic resources and TK). While Canada is not obligated to legislate to provide fair and equitable ABS under the CBD, the existing legal framework requires that researchers consult and partner with Aboriginal communities and to consider their interests. Canada's main funding agencies have policies on research with Aboriginal partners and recommend research agreements (8,64,65). However, they provide inadequate guidance on how to structure such agreements to achieve mutual benefit, reflect unequal bargaining power, and/or account for the long-term relationships between parties.

Relational contract theory may offer a useful framework to address the social, cultural and legal hurdles inherent in creating research agreements. This theory takes into account many of the struggles that research partners have while trying to develop mutually beneficial partnerships including power imbalances, expectations of the other party, ability to adjust to changing circumstances, and respect of the other while pursuing individual goals. The norms of relational contract theory provide guidance to parties negotiating research agreements that promote mutual benefit in research partnerships. Despite the difficulties, some research partnerships between northern communities and researchers exist that reflect many of these norms. McGill University researchers and the Mohawk community of Kahnawake jointly developed a Code of Ethics for The Kahnawake Schools Diabetes Prevention Project. This code clearly outlines procedures for the control of data and dissemination of results, including how to handle disagreements and conflicts (66). An empirical analysis of such agreements, combined with discussions with the parties about the extent to which their interests and expectations are reflected over the long term, will add to our understanding of best-practice. Such analysis will enable the drafting of research agreements that promote fair and equitable access to and benefits sharing of genetic resources and associated TK.

## Footnotes

1 Article 2 of the Convention on Biological Diversity defines genetic resources as “genetic material of actual or potential value” and genetic material is defined as “any material of plant, animal, microbial or other origin containing functional units of heredity” (10).

2 Intellectual property includes artistic expressions, formulas and trademarks and is protected through patent, copyright, trademark and similar laws.
